# Quantification of Bevacizumab Activity Following Treatment of Patients With Ovarian Cancer or Glioblastoma

**DOI:** 10.3389/fimmu.2020.515556

**Published:** 2020-10-15

**Authors:** Christophe Lallemand, Rosa Ferrando-Miguel, Michael Auer, Sarah Iglseder, Theresa Czech, Anouk Gaber-Wagener, Franziska Di Pauli, Florian Deisenhammer, Michael G. Tovey

**Affiliations:** ^1^Svar Life Science France, Villejuif, France; ^2^Department of Neurology, Innsbruck Medical University, Innsbruck, Austria; ^3^Department of Gynecology, Innsbruck Medical University, Innsbruck, Austria

**Keywords:** bevacizumab, ADCC, glioblastoma, ovarian cancer, VEGF, bioassay, reporter gene assay

## Abstract

Highly sensitive reporter-gene assays have been developed that allow both the direct vascular endothelial growth factor (VEGF) neutralizing activity of bevacizumab and the ability of bevacizumab to activate antibody dependent cellular cytotoxicity (ADCC) to be quantified rapidly and in a highly specific manner. The use of these assays has shown that in 46 patients with ovarian cancer following four cycle of bevacizumab treatment, and in longitudinal samples from the two patients that respond to bevacizumab therapy from a small cohort of patients with glioblastoma, that there is a reasonably good correlation between bevacizumab drug levels determined by ELISA and bevacizumab activity, determined using either the VEGF-responsive reporter gene, or the ADCC assays. One of the two primary non-responders with glioblastoma exhibited high levels of ADCC activity suggesting reduced bevacizumab Fc engagement *in vivo* in contrast to the other primary non-responder, and the two secondary non-responders with a decreasing bevacizumab PK profile, determined by ELISA that exhibited low to undetectable ADCC activity. Drug levels were consistently higher than bevacizumab activity determined using the reporter gene assay in serial samples from one of the secondary non-responders and lower in some samples from the other secondary non-responder and ADCC activity was markedly lower in all samples from these patients suggesting that bevacizumab activity may be partially neutralized by anti-drug neutralizing antibodies (NAbs). These results suggest that ADCC activity may be correlated with the ability of some patients to respond to treatment with bevacizumab while the use of the VEGF-responsive reporter-gene assay may allow the appearance of anti-bevacizumab NAbs to be used as a surrogate maker of treatment failure prior to the clinical signs of disease progression.

## Introduction

The anti-vascular endothelial growth factor-A (VEGFA) monoclonal antibody bevacizumab (Avastin^®^) is used extensively to treat recurrent disease in patients with ovarian cancer and patients with glioblastoma who have failed first line therapy (1–3). Although, bevacizumab treatment results in a high initial response rate the effect is transient and most patient’s tumors eventually progress (2, 3). The mechanisms of bevacizumab treatment failure are poorly understood (1) and an accurate assessment of the treatment response in individual patients is key to better understand the most effective means of optimizing bevacizumab treatment.

Current methods for quantifying the activity of human VEGF, or antibodies that neutralize its activity, such as bevacizumab, are bioassays based on the ability of anti-VEGF antibodies to inhibit the proliferation or migration of primary human umbilical vein endothelial cells (HUVEC) or other cells expressing VEGFR receptors, following treatment of the cells with VEGF (4). Such assays can take several days to perform, are subject to a high degree of variation, and are difficult to validate. Reporter-gene assays based on the establishment of a stable cell line transfected with a luciferase reporter-gene placed under the control of a drug responsive chimeric promoter, provide highly sensitive and reproducible methods for quantifying drug activity (5–7) and although a VEGF-specific reporter gene assay has been developed previously (8) there is a need for an assay with improved sensitivity and dynamic range. Highly sensitive reporter gene assays are described herein that allow both the direct VEGF neutralizing activity of bevacizumab to be quantified with improved sensitivity and the ability of bevacizumab to activate antibody dependent cellular cytotoxicity (ADCC) to be quantified rapidly and in a highly specific manner. These assays have been used to better understand the action of bevacizumab in 46 patients with ovarian cancer following analysis of samples taken after four cycle of bevacizumab treatment and in longitudinal samples from in a small cohort of patients with glioblastoma presenting different types of response to treatment with bevacizumab.

## Materials and Methods

### VEGF Responsive Reporter Cells

Human embryonic kidney HEK293 cells (ATCC Catalogue N° CRL1573) were transfected with a 5-fold tandem repeat of the upstream activation sequence (UAS), cCGGAGGACTGTCCTCCGagtc, of gal-4 regulating transcription of the firefly luciferase (FL) reporter gene. The cells were also co-transfected with an expression vector encoding a chimeric transcription factor consisting of the gal4 DNA binding domain (nt:1–130) fused to the *trans*-activating domain of Elk-1 (nt:307–427), together with an expression vector for human VEGFR2. A clonal cell line was established that exhibited a high degree of VEGF responsiveness following treatment of the VEGF-responsive cells with increasing concentrations of VEGFA for 18 h at 37°C prior to quantification of VEGF-induced FL activity using the Bright-Glo^®^ (Promega, Madison, WI, United States) reagent and a GloMax^®^ luminometer (Promega, Madison, WI, United States).

### Quantification of Bevacizumab Activity

A fixed dilution or serial dilutions of the sample or bevacizumab standard to be tested were incubated for 30 min at 37°C with 25 ng/mL of VEGF. The samples are then incubated with the VEGF responsive reporter-gene cells for 18 h at 37°C prior to quantification of VEGF-induced FL activity as described in the preceeding section.

### Quantification of ADCC Activity

The novel engineered Jurkat ADCC effector cells expressing the V-158 wild-type variant of the FcγRIIIA receptor (CD16a) and the FL reporter-gene under the control of the principal transcription factors involved in FcγRIIIA signal transduction has been described previously (9). VEGF(−) target cells derived from human embryonic HEK293 cells, that do not express detectable levels of VEGF (Lallemand, unpublished results), were transfected with the costimulatory molecules CD80 and CD86 that were found to enhance the dynamic range of the ADCC assay for several different therapeutic monoclonal antibodies and the appropriate antigen positive target cells (Lallemand, unpublished results). VEGF (−) target cells expressing the costimulatory molecules CD80 and CD86 were again found not to express detectable levels of VEGF or ADCC activity in the presence of bevacizumab and the ADCC effector cells. The VEGF (++) target cells that express membrane-bound non-cleavable VEGFA were established by transfecting VEGF (−) target cells expressing the costimulatory molecules CD80 and CD86 with the gene encoding codon-optimized VEGFA fused to the coding sequence of the transmembrane region of TNFα bearing a mutation in the protease cleavage site as described previously (7).

### Quantification of Bevacizumab Serum Concentrations by Capture ELISA

Microtiter plates were coated overnight with human recombinant VEGF_165_ (R&D systems, catalog# 293-VE-001MG/CF) at a concentration of 0.15 μg/ml followed by blocking for 2 h with 3% BSA. For the standard curve recombinant bevacizumab (Avastin^®^) was used at concentrations ranging from 7 to 500 μg/ml. Test samples (diluted 1:100 in 0.1% blocking buffer) including negative controls were added and incubated for 1 h at 37°C. All wells, including test samples and standard curves, were then incubated with 100 μl of a monoclonal HRP-conjugated anti-bevacizumab antibody diluted 1:5000 (AbD Serotec, catalog# HCA 184P). Finally, ortho-phenylenediamine HRP substrate was added and the reaction was stopped with hydrochloric acid. Plates were washed four times with 0.05% PBS-TWEEN between each step. Plates were read at 492 nm and again at 620 nm and serum concentrations were determined using the standard curve. All procedures were optimized in the preceding assay validation steps.

### Patient Population

A cohort of 46 patients with ovarian cancer and a second cohort of 6 patients diagnosed with glioblastoma according to the World Health Organization classification scheme (9) were both hospitalized at the Innsbruck University Hospital. Patients with glioblastoma were classified according to clinical and/or radiological outcomes as responders, who presented no tumor progression, primary non-responders presenting no response at all, and secondary non-responders, who exhibited an objective response followed by tumor progression. Patients were included in the study following written informed consent in accordance with the institution guidelines. This study was approved by the ethics committee of the University of Innsbruck: Approval number EKNR 1054/2017.

## Results

**FIGURE 1:**
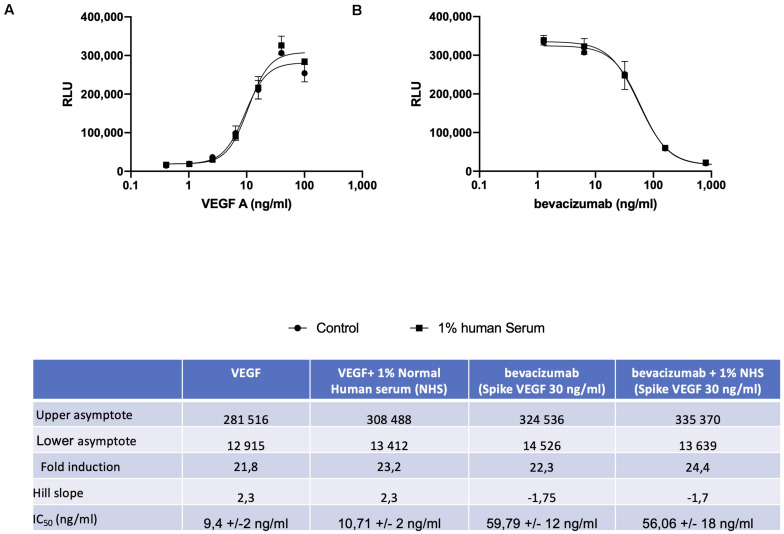
The VEGF responsive reporter-gene cell line was incubated for 18 h with increasing concentrations of VEGFA either alone or in the presence of 1.0% normal human serum prior to quantification of FL activity as described in the section “Materials and Methods” **(A)**. Increasing concentrations of bevacizumab were mixed with 25 ng/ml of VEGFA for 30 min at room temperature prior to incubation for 18 h with VEGF responsive reporter-gene cells either alone or in the presence of 1.0% normal human serum and quantification of FL activity as described in the section “Materials and Methods” **(B)**. The associated Table to the Figure shows the principal parameters of a 4PL plot determined using the Prism software.

**FIGURE 2:**
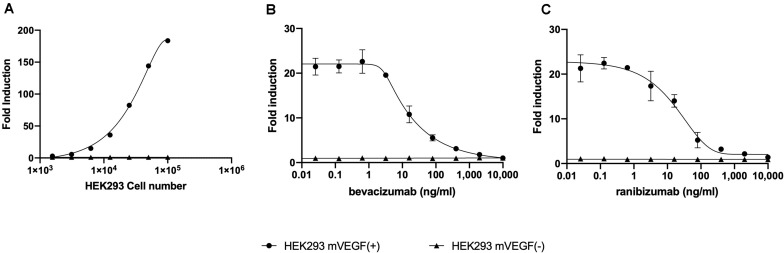
Increasing concentrations of HEK293 mVEGF (+) target cells were incubated with 2.5 × 10^4^ of the VEGF responsive reporter-gene cell line for 18 h at 37°C prior to the quantification of FL activity as described in the section “Materials and Methods” **(A)**. Increasing concentration bevacizumab **(B)** or ranibizumab **(C)** were mixed with 10,000 mVEGF (+) target cells, sufficient to give an approximately 20-fold increase in the FL response of the VEGF responsive reporter-gene cell line, or 10,000 mVEGF (−) target cells for 30 min at room temperature prior to incubation for 18 h at 37°C with the VEGF responsive reporter-gene cell line and quantification of FL activity as described in the section “Materials and Methods”.

**FIGURE 3:**
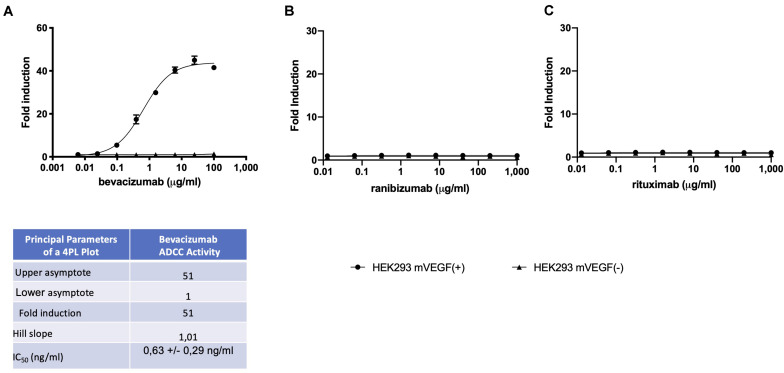
ADCC effector cells (E) at a concentration of (1.2 × 10^5^ cells/well), were incubated with mVEGF (+) or mVEGF(−) target cells (T) at an E:T ratio of 3:1 and increasing concentrations of bevacizumab **(A)** ranibizumab **(B)**, or rituximab **(C)** for 4 h prior to the quantification of FL activity as described in the section “Materials and Methods”. The associated Table to the Figure shows the principal parameters of a 4PL plot of fold-induction of the ADCC activity of bevacizumab determined using the Prism software. To eliminate possible non-specific effects, the ADCC activity of each serum sample was first determined by interpolation of the standard curve of bevacizumab activity determined using both mVEGF (+) and mVEGF(−) target cells and the values obtained in the presence of the mVEGF(−) target cells were subtracted from those obtained using the mVEGF (+) terget cells and using the Prism software (GraphPad, France).

A reporter-gene cell line expressing FL under the control of an Elk-1-responsive chimeric promoter, one of the principal transcription factors involved in the VEGF signal transduction pathway (10), was developed that responds specifically to treatment of cells with VEGF (Figure 1A and Supplementary Figure S1). The reporter-gene cell line was used for the quantification of bevacizumab activity based on the ability of bevacizumab to neutralize soluble VEGFA as reflected by an inhibition of VEGF-induced FL activity (Figure 1B). The bevacizumab reporter-gene cell line described herein exhibited improved characteristics, including a dynamic range of approximately 20-fold and a IC_50_ of approximately 60 ng/ml (Insert to Figure 1) relative to that published previously for a bevacizumab responsive reporter-gene assay based on a NFAT responsive promoter (3), and exhibited a stable response over an extended number of passages in the presence of the selective agents (Supplementary Figure S2). The intra-assay and inter-assay precision for the bevacizumab reporter-gene cell line described herein ranged from 6 to 17% and 5 to 11%, respectively, for the coefficients of variation for the principal parameters of a 4PL plot (Insert to Supplementary Figure S3). The accuracy of the assay, determined from the ratio of the measured to the expected values for concentrations of bevacizumab ranging from 30 to 150 % of the expected values yielded a linear curve with a *R*^2^ of 0.963 (Supplementary Figure S4). The presence of normal human serum was without effect on the assay results when tested at final concentrations ranging from 2.5 to 10.0 % (Supplementary Figure S5) encompassing the final concentration of human serum present in patient samples analyzed (Figure 1). The ability of the VEGF-responsive reporter-gene cell line to quantify the activity of membrane-bound non-cleavable VEGFA (Figure 2A) led to the observation that bevacizumab is able to neutralize membrane bound VEGF and provides a means of quantifying this activity using the mVEGF (+) target cells that express membrane-bound non-cleavable VEGF2A (Figure 2B). In contrast, bevacizumab did not exhibit any effect on FL reporter-gene activity in the presence of mVEGF (−) target cells (Figure 2B). Ranibizumab (Lucentis^®^), a derivative of bevacizumab lacking a Fc receptor was also shown to neutralize membrane bound VEGFA in the presence of mVEGF (+) but not mVEGF (−) target cells (Figure 2C). The ability of bevacizumab to activate ADCC was also quantified using an effector cell line expressing the FcγRIIIa receptor (CD16) that responds specifically to binding of the Fc moiety of an antibody to the FcγRIIIa receptor by activation of the FL reporter-gene (7) in the presence of target cells that express non-cleavable membrane bound VEGFA (Figure 3A). No ADCC activity was observed when bevacizumab was tested in the presence of target cells that do not express membrane bound VEGFA (Figure 3A) or when ranibizumab (Lucentis^®^), a derivative of bevacizumab lacking a Fc moiety, was tested in the presence of target cells that express non-cleavable membrane bound VEGFA (Figure 3B) or when the effector cell line expressing the FcγRIIIa receptor and target cells that express non-cleavable membrane bound VEGFA were treated with increasing concentrations of the anti-CD20 antibody rituximab (Figure 3C).

**FIGURE 4:**
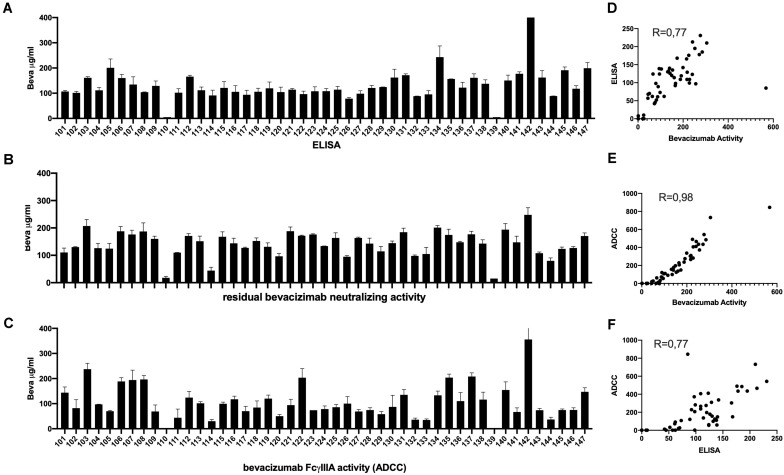
Serum samples from 46 patients with ovarian cancer described in Supplementary Table S1 were tested after four cycles of bevacizumab treatment for the presence of circulating levels of bevacizumab determined by ELISA (Panel A), and for the ability of bevacizumab to neutralize VEGF (Panel B), and to activate ADCC activity (Panel C) as described in the section “Materials and Methods.” To eliminate possible non-specific effects, the ADCC activity of each serum sample was first determined using both mVEGF (+) and mVEGF(−) target cells and the values obtained in the presence of the mVEGF(−) target cells were subtracted from those obtained using the mVEGF (+) and the final results were determined by interpolation of the standard curve of bevacizumab activity (Figure 3, Panel A) using the Prism software (GraphPad, France). The values obtained for the circulating levels of bevacizumab determined by ELISA were compared with the values obtained for the neutralization of VEGF activity (Panel D) or the values obtained for the ADCC activity of bevacizumab were compared with the values obtained for the neutralization of VEGF activity (Panel E) or the circulating levels of bevacizumab determined by ELISA (Panel F).

**FIGURE 5:**
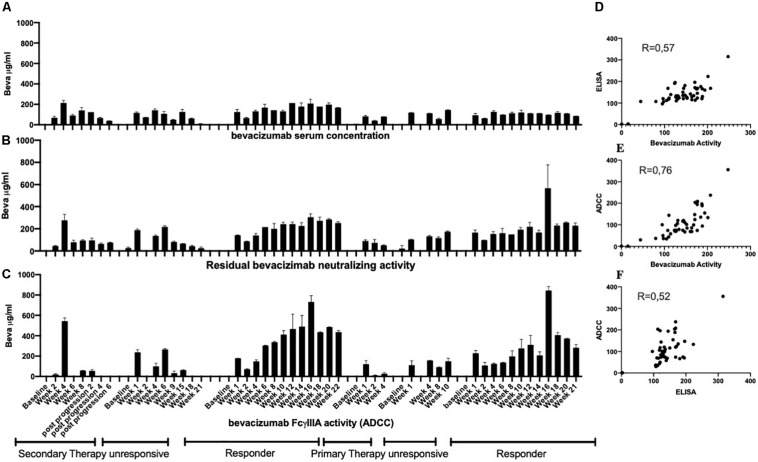
Serial serum samples from the patient cohort described in Table 1 were tested for the presence of circulating levels of bevacizumab using an ELISA (Panel A) as described in the section “Materials and Methods.” The same samples were also tested for bevacizumab VEGF neutralizing activity (Panel B) and bevacizumab ADCC activity (Panel C) as described in the section “Materials and Methods.” To eliminate possible non-specific effects, the ADCC activity of each serum sample was first determined using both mVEGF (+) and mVEGF(−) target cells and the values obtained in the presence of the mVEGF(−) target cells were subtracted from those obtained using the mVEGF (+) and the final results were determined by interpolation of the standard curve of bevacizumab activity (Figure 3, Panel A) using the Prism software (GraphPad, France). The values obtained for the circulating levels of bevacizumab determined by ELISA were compared with the values obtained for the neutralization of VEGF activity (Panel D) or the values obtained for the ADCC activity of bevacizumab were compared with the values obtained for the neutralization of VEGF activity (Panel E) or the circulating levels of bevacizumab determined by ELISA (Panel F).

Analysis of serum samples from a cohort of 46 patients with ovarian cancer following four cycles of bevacizumab treatment revealed (Supplementary Table S1) a reasonably good correlation between circulating drug levels determined by ELISA and the VEGFA neutralizing activity of bevacizumab (Figures 4A,B) and a close correlation between the VEGF2A neutralizing activity of bevacizumab and the ability of bevacizumab to activate ADCC (Figures 4B,C). Analysis of the results using the nonparametric Spearman rank correlation coefficient give a ρ value of 0.77 between circulating drug levels determined by ELISA and the VEGF2A neutralizing activity of bevacizumab (Figure 4D), a ρ value of 0.98 between the ability of bevacizumab to activate ADCC and the VEGFA neutralizing activity of bevacizumab (Figure 4E), and a ρ value of 0.77 between the ability of bevacizumab to activate ADCC and circulating drug levels of bevacizumab determined by ELISA (Figure 4F). Analysis of longitudinal samples from a small cohort of patients diagnosed with glioblastoma (Table 1) according to the World Health Organization classification scheme (9) and presenting different types of response to treatment with bevacizumab, showed that overall, bevacizumab neutralizing activity correlated reasonably well with bevacizumab protein levels in most samples from patients irrespective of whether they were classified as responders, primary non-responders, or secondary non-responders although there was a tendency toward lower levels of bevacizumab neutralizing activity in samples from the two secondary non-responders (Figures 5A,B). A closer correlation was observed between the VEGFA neutralizing activity of bevacizumab and the ability of bevacizumab to activate ADCC (Figures 5B,C). In contrast, the host mediated ADCC activity of bevacizumab did not appear to be correlated solely with the level of circulating bevacizumab. Thus, the ADCC activity of bevacizumab was relatively low in most samples from the two patients classified as responders, relatively high in one of the two primary non-responders, and low to barely detectable in the other patient classified as a primary non-responder and the two secondary non-responders (Figure 5). Analysis of the results using the nonparametric Spearman rank correlation coefficient give a ρ value of 0.57 between circulating drug levels determined by ELISA and the VEGFA neutralizing activity of bevacizumab (Figure 5D), a ρ value of 0.76 between the ability of bevacizumab to activate ADCC and the VEGFA neutralizing activity of bevacizumab (Figure 5E), and a ρ value of 0.52 between the ability of bevacizumab to activate ADCC and circulating drug levels determined by ELISA (Figure 5, Panel F). We have shown that bevacizumab, but not the TNFα antagonist infliximab, also exhibits marked ADCC activity against human glioblastoma cells *in vitro* (Supplementary Figure S6).

**TABLE 1:** Patients with glioblastoma: Treatment schedule*.

**Patient**	**Sex**	**Age**	**Recent or current use of corticosteroids**	**Temodal**	**Radiotherapy**	**Other previous chemo-therapy**	**Clinical response**
IBK 1	F	38	Yes	Yes	Yes	No	Primary therapy unresponsive
IBK 2	M	61	Yes	Yes	Yes	No	Secondary therapy unresponsive
IBK 3	M	50	Yes	Yes	Yes	No	Primary therapy unresponsive
IBK 5	F	56	No	Yes	Yes	No	Responder
IBK 7	F	57	Yes	Yes	Yes	No	Responder
IBK 17	F	38	No	Yes	Yes	No	Secondary therapy unresponsive

*All patients with glioblastoma were treated with 15 mg of bevacizumab per kg of body weight every 3 weeks in addition to Temodal and Radiotherapy.

## Discussion

Bevacizumab is used to target VEGF-dependent angiogenesis in patients with platinum-sensitive recurrent ovarian cancer (3) and is also used extensively to treat recurrent disease in patients with glioblastoma who have failed first line therapy (1, 2). Although bevacizumab treatment results in a high initial response rate the results are transient and most patient’s tumors eventually progress (1–3). High grade glioblastomas produce large quantities of VEGFA that stimulates the proliferation of endothelial cells leading to the development of an abnormal vasculature (3). Bevacizumab is thought to act in part by reducing tumor-induced vascularization thereby limiting tumor growth (10). VEGFA exists in multiple isoforms, as a result of both alternative splicing of exons 6 and 7 and proteolysis, and the most common isoform VEGFA_165_ is also present in a soluble form and as part of the extracellular matrix (11). The ability to quantify the activity of membrane bound VEGFA in addition to soluble VEGF as shown herein may facilitate a better understanding of the action of bevacizumab on tumor-induced vascularization in both gynecologic and neurologic tumors. In addition to reducing tumor-induced vascularization bevacizumab is also thought to exert direct anti-tumor activity against gliomas that express VEGF on their cell surface (12–15) and in animal models of ovarian cancer (16) and again the ability to quantify the activity of membrane bound VEGFA may also shed light on this process. There is also evidence to suggest that bevacizumab increases the sensitization of tumor cells to cytotoxic agents (13) and the ability to quantify the effect of bevacizumab on both soluble VEGF and membrane bound VEGF may also help elucidate the mechanisms of this process. Bevacizumab activity, determined using a VEGF responsive reporter-gene assay, correlated reasonably well with circulating levels of bevacizumab determined by ELISA in serial samples from the two patients classified as responders and were generally lower in serial samples from the two patients classified as secondary non-responders suggesting that treatment failure in secondary non-responders may be attributed at least in part to the presence of neutralizing anti-bevacizumab antibodies that could arise during bevacizumab treatment. Furthermore, the activity of circulating bevacizumab as determined by its ability to activate antibody-dependent cellular cytotoxicity was very low to undetectable in the serial samples from the two secondary non-responders. The microenvironment of glioblastomas contains numerous innate immune cells including microglia-macrophages and other immune cells resulting from alterations in the blood-brain barrier in addition to tumor cells (15). Studies using mouse models suggest that VEGF blockage can lead to an increased recruitment of monocytes as well as changes in dendritic cell sub-sets that may alter the adaptive immune response to the tumor (16). The anti-tumor activity of numerous monoclonal antibodies is mediated in part by the stimulation of cellular immunity (17) such as ADCC and antibody dependent cellular phagocytosis (ADCP). The two patients classified as responders both exhibited readily detectable levels of ADCC activity that overall correlated reasonably well with circulating levels of bevacizumab protein determined by ELISA and bevacizumab activity determined using the VEGF-responsive reporter-gene assay. To our knowledge this is the first report that bevacizumab exhibits ADCC activity both *in vitro*, including against human glioblastoma target cells, and in samples of serum from patients. No ADCC activity was observed when bevacizumab was tested in the presence of target cells that do not express membrane bound non-cleavable VEGFA or when a derivative of bevacizumab lacking a Fc receptor (ranibizumab, Lucentis^®^) was tested in the presence of target cells that express membrane bound VEGFA, attesting to the validity of the results. Remarkably, a high level of bevacizumab ADCC activity was observed in one patient classified as a primary non-responder but not in samples from the other patients also classified as a primary non-responder or the two patients classified as secondary non-responder. Although it is well established that the neutralizing antibody response to the variable region of therapeutic antibodies can limit their efficacy (18) our results suggest that for antibodies that act in part by the activation of cellular immunity an immune response the to Fc moiety of the antibody may also limit their efficacy. Although it is difficult to draw any firm conclusions from these results due to the restricted number of samples tested from two small cohorts of patients included in this pilot study, they do show that it is indeed possible to quantify both the direct VEGF neutralizing activity of bevacizumab and the host mediated ADCC of activity bevacizumab in samples from patients with ovarian cancer or glioblastoma treated with bevacizumab and that clear differences are observed between samples from individual patients.

Although bevacizumab treatment was found not to confer an increase in overall survival in newly diagnosed patients with glioblastoma nor to confer an overall survival advantage in combination therapy (19), bevacizumab treatment can prolong progression-free survival and is used extensively in routine clinical practice in the United States and some other countries (20). An accurate assessment of the treatment response in individual patients is key to a better understanding of the most effective means of optimizing bevacizumab treatment.

## Data Availability Statement

All datasets generated for this study are included in the article/Supplementary Material.

## Ethics Statement

The studies involving human participants were reviewed and approved by Geschäftsstelle der Ethikkommission der Medizinischen Universität Innsbruck Innrain 43 A-6020 Innsbruck. The patients/participants provided their written informed consent to participate in this study.

## Author Contributions

CL and RF-M performed the VEGF reporter-gene and ADCC assay and analysis of the data under the direction of MT who also wrote the manuscript. MA, SI, TC, AG-W, FDP, and FD supervised the clinical study. MA and SI performed the PK analysis under the direction of FD. All authors contributed to the article and approved the submitted version.

## Conflict of Interest

CL, RF-M, andMTwere employed by Svar life Science France. The remaining authors declare that the research was conducted in the absence of any commercial or financial relationships that could be construed as a potential conflict of interest.

## Supplementary Material

FIGURE S1 Illustration of the interaction of VEGFA with its cell-surface receptor and activation of the cytoplasmic signal transduction pathway resulting in phosphorylation and activation of the chimeric Elk-1::Gal4 transcription factor in the nucleus. The chimeric transcription factor consisting of the transactivating domain of Elk-1 fused to the DNA binding domain of Gal4 then binds to the 5-fold tandem repeat of the Gal4 upstream activation sequence (UAS) resulting in activation of the firefly luciferase reporter-gene.

FIGURE S2 The stability of the VEGF responsive reporter-gene cell line was determined by testing VEGFA induced FL activity at regular intervals for 20 passages by incubating the VEGF responsive reporter-gene cells for 18 h with increasing concentrations of VEGFA prior to quantification of FL activity as described in the section “Materials and Methods”.

FIGURE S3 Inter and intra-assay precision was determined by incubating increasing concentrations of bevacizumab with 4 individual samples of VEGFA at a final concentration of 25 ng/ml for 30 min at room temperature on a single microtiter plate **(A)** or with a single sample of VEGFA at a final concentration of 25 ng/ml and incubated for 30 min at room temperature on 4 individual microtiter plates **(B)** prior to incubation for 18 h at 37°C with the VEGF responsive reporter-gene cell line and quantification of FL activity as described in the section “Materials and Methods.” The associated Table to Figure 2 shows the principal parameters of a 4PL plot determined using the Prism software together with intra-plate or inter-plate percentage coefficients of variation (% CV).

FIGURE S4 The accuracy and linearity of the reporter-gene assay was determined by incubating concentrations bevacizumab corresponding to 25, 50, 100, 125, and 150% of the expected value with VEGFA at a final concentration of 25 ng/ml for 30 min at room temperature prior to incubation for 18 h at 37°C with the VEGF responsive reporter-gene cell line and quantification of FL activity as described in the section “Materials and Methods”.

FIGURE S5 Increasing concentrations of bevacizumab were mixed with 25 ng/ml of VEGFA for 30 min at room temperature prior to incubation for 18 h with VEGF responsive reporter-gene cells either alone or in the presence of 2.5, 5, or 10% normal human serum and quantification of FL activity as described in the section “Materials and Methods” **(B)**. The associated Table to Figure 1 shows the principal parameters of a 4PL plot determined using the Prism software.

FIGURE S6 ADCC effector cells (E) at a concentration of (1.2 × 10^5^ cells/well), were incubated with human U87 glioblastoma target cells (T) at an E:T ratio of 3:1 and 100 μg/ml of bevacizumab or 100 μg/ml of inflixumab for 4 h prior to the quantification of ADCC activity as described in the section “Materials and Methods”.

TABLE S1 Patients with ovarian cancer: Treatment schedule*.

## References

[1] Li Y Clarke J Cha S. Bevacizumab in recurrent glioma: patterns of treatment failure. *Brain Tumor Res Treat.* (2017) 5:1–9. 10.14791/btrt.2017.5.1.1 28516072 PMC5433944

[2] Wenger KJ Wagner M You SJ Franz K Harter PN Burger MC Bevacizumab as last-line treatment for glioblastoma following failure of radiotherapy, temozolomide and lomustine. *Oncol Lett.* (2017) 14:1141–6. 10.3892/ol.2017.6251 28693286 PMC5494648

[3] Garcia J Hurwitz HI Sandler AB Miles D Coleman RL Deurloo R Vevacizumab (Avastin^®^) in cancer treatment: a review of 15 years of clinical experience and future outlook. *Cancer Treat Rev.* (2017) 86:102017. 10.1016/j.ctrv.2020.102017 32335505

[4] Wang A Fei D Vanderlaan M Song A. Biological activity of bevacizumab, a humanized anti-VEGF antibody in vitro. *Angiogenesis.* (2004) 7:335–45. 10.1007/s10456-004-8272-2 15886877

[5] Parekh BS Berger E Sibly S Cahya S Xiao L LaCerte MA Development and validation of an antibody-dependent cell-mediated cytotoxicity-reporter-gene assay. *MAbs.* (2012) 4:310–8. 10.4161/mabs.19873 22531445 PMC3355484

[6] Lallemand C Kavrochorianou N Steenholdt C Bendtzen K Ainsworth MA Meritet JF Reporter gene assay for the quantification of the activity and neutralizing antibody response to TNFα antagonists. *J Immunol Methods.* (2011) 373:229–39. 10.1016/j.jim.2011.08.022 21910993

[7] Lallemand C Liang F Staub F Simansour M Vallette B Huang L A novel system for the quantification of the ADCC activity of therapeutic antibodies. *J Immunol Res.* (2017) 10:1–19. 10.1155/2017/3908289 29104875 PMC5635472

[8] Wang L Xu GL Gao K Wilkinson J Zhang F Yu L Development of a robust reporter-based assay for the bioactivity determination of anti-VEGF therapeutic antibodies. *J Pharm Biomed Anal.* (2016) 125:212–8. 10.1016/j.jpba.2016.03.042 27042807

[9] Louis DN Ohgaki H Wiestler OD Wk C. WHO classification of tumours of the central nervous system. *Acta Neuropathol.* (2016) 6:803–20. 10.1007/s00401-016-1545-1 27157931

[10] Schweighofer B Schultes J Pomyje J Hofer E. Signals and genes induced by angiogenic growth factors in comparison to inflammatory cytokines in endothelial cells. *Clin Hemorheol Microcirc.* (2007) 37:57–62. 17641395 PMC3103851

[11] Ferrara N Adams AP. Ten years of anti-vascular endothelial growth factor therapy. *Nat Rev Drug Ther.* (2016) 15:385–403. 10.1038/nrd.2015.17 26775688

[12] Kargiotis O Rao JS Kyritsis AP. Mechanisms of angiogenesis in gliomas. *J Neurooncol.* (2006) 78:281–93. 10.1007/s11060-005-9097-6 16554966

[13] Ellis LM Hicklin DJ. VEGF-targeted therapy: mechanisms of anti-tumour activity. *Nat Rev Cancer.* (2008) 8:579–91. 10.1038/nrc2403 18596824

[14] Thompson EM Frenkel EP Neuwelt EA. The paradoxical effect of bevacizumab in the therapy of malignant gliomas. *Neurology.* (2011) 76:87–93. 10.1212/WNL.0b013e318204a3af 21205697 PMC3030223

[15] Charles NA Holland EC Gilbertson R Glass R Kettenmann H. The brain tumor microenvironment. *Glia.* (2012) 60:502–14. 10.1002/glia.21264 22379614

[16] Souberan A Brustlein S Couarne G Chasson L Tchoghandjian A. Effects of VEGF blockade on the dynamics of the inflammatory landscape in glioblastoma-bearing mice. *J Neuroinflammation.* (2019) 16:191–206. 10.1186/s12974-019-1563-8 31660979 PMC6816183

[17] Marcucci F Bellone M Rumio C Corti A. Approaches to improve tumor accumulation and interactions between monoclonal antibodies and immune cells. *MAbs.* (2013) 5:34–46. 10.4161/mabs.22775 23211740 PMC3564884

[18] Steenholdt C Bendtzen K Brynskov J Thomsen OØ Ainsworth MA. Cut-off levels and diagnostic accuracy of infliximab trough levels and anti-infliximab antibodies in Crohn’s disease. *Scand J Gastroenterol.* (2011) 46:310–8. 10.3109/00365521.2010.536254 21087119

[19] Chinot OL Wick W Mason W Henriksson R Saran F Nishikawa R Bevacizumab plus adiotherapy/temozolomide for newly diagnosed glioblastoma. *N Eng J Med.* (2014) 370:709–22. 10.1056/NEJMoa1308345 24552318

[20] Wick W Osswail M Wick A Winkler F. Treatment of glioblastoma in adults. *Ther Adv Neurol Disord.* (2018) 11:1756286418790452. 10.1177/1756286418790452 30083233 PMC6071154

