# Supplementary figures and images for: Quantification of Bevacizumab Activity Following Treatment of Patients With Ovarian Cancer or Glioblastoma
Source: Front Immunol. 2020 Oct 15;11:515556. doi: 10.3389/fimmu.2020.515556 (PMC7593583; doi:10.3389/fimmu.2020.515556)

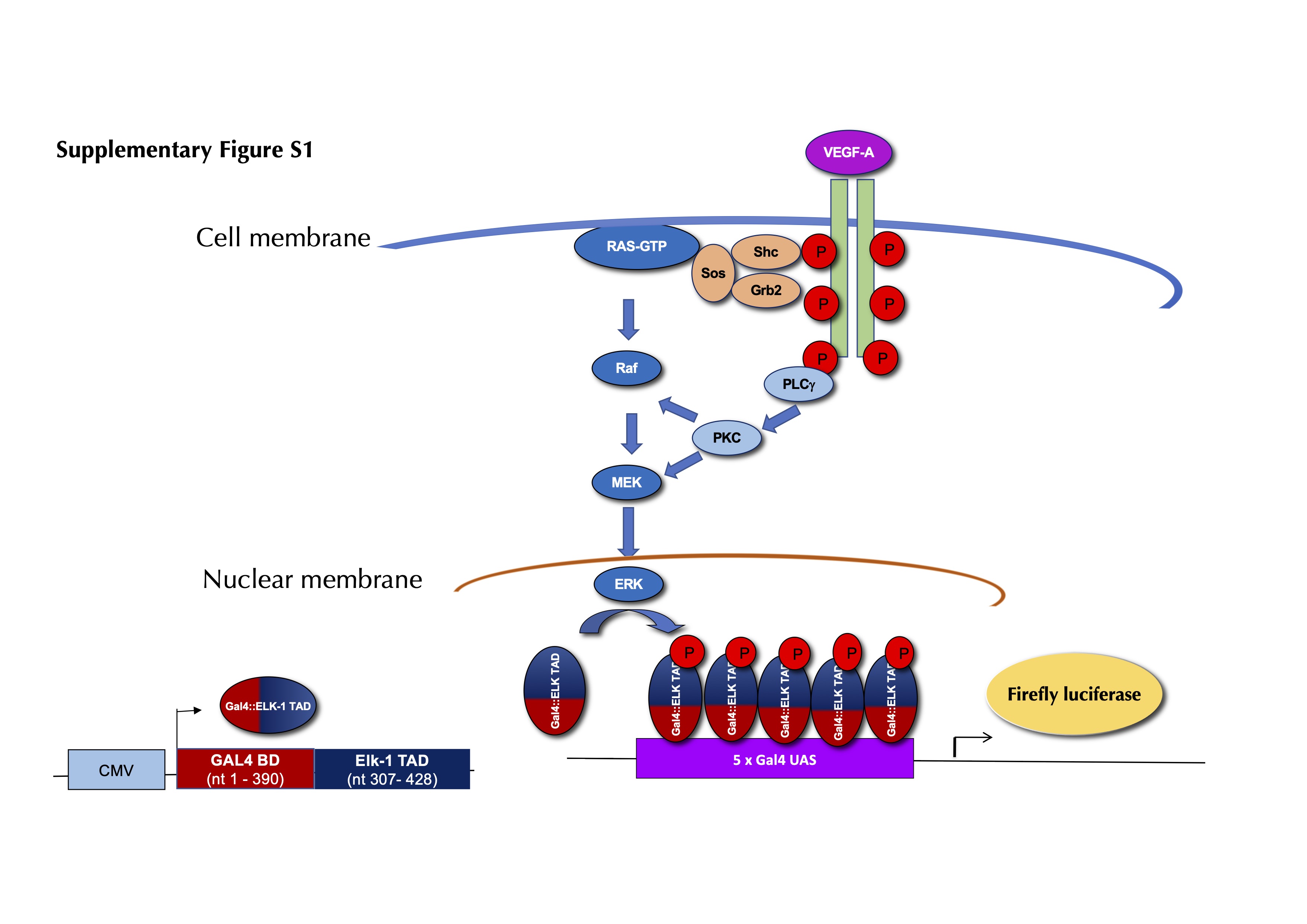

Supplement: FIGURE S1 — Illustration of the interaction of VEGFA with its cell-surface receptor and activation of the cytoplasmic signal transduction pathway resulting in phosphorylation and activation of the chimeric Elk-1::Gal4 transcription factor in the nucleus. The chimeric transcription factor consisting of the transactivating domain of Elk-1 fused to the DNA binding domain of Gal4 then binds to the 5-fold tandem repeat of the Gal4 upstream activation sequence (UAS) resulting in activation of the firefly luciferase reporter-gene. [file Image_1.JPEG]

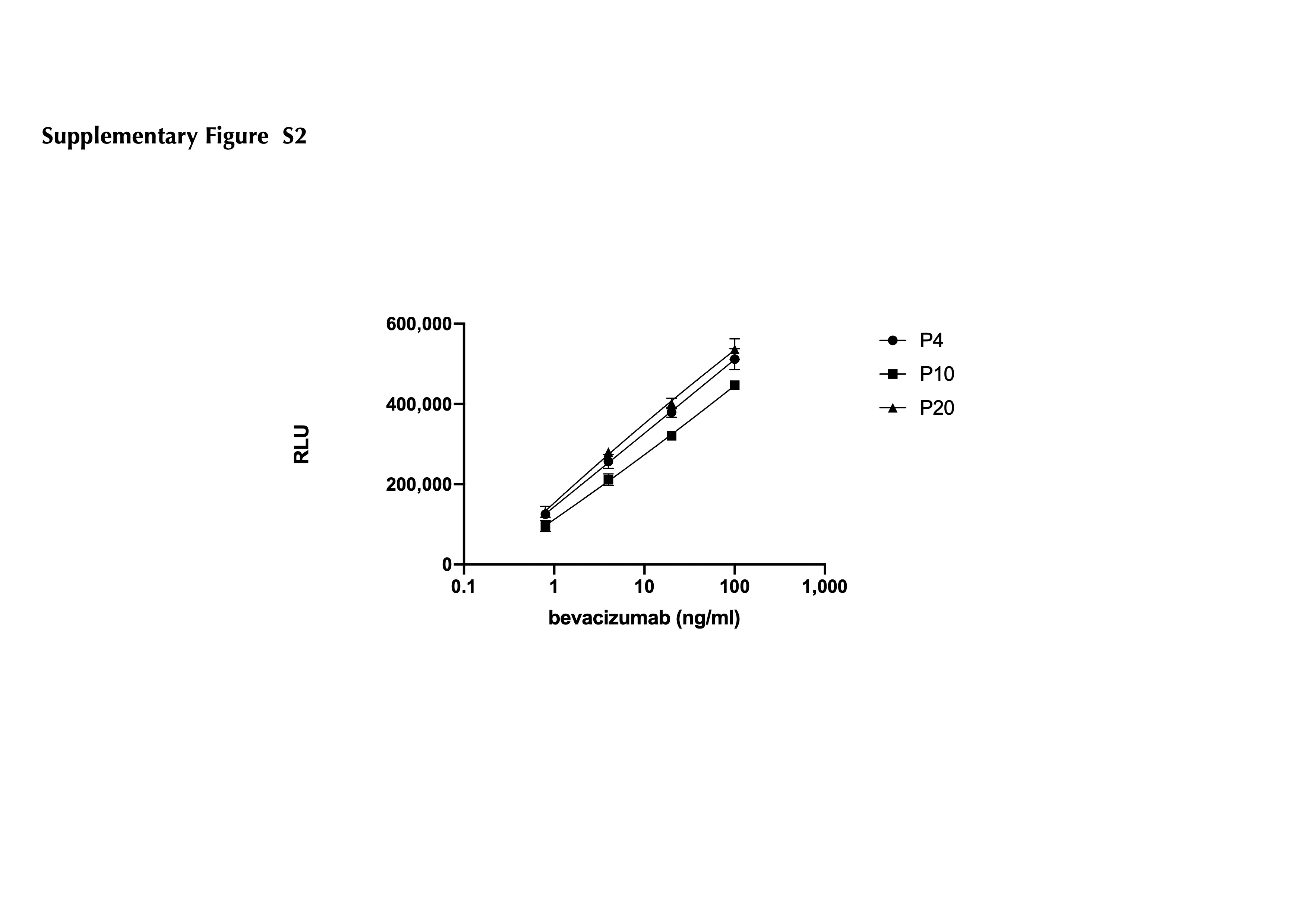

Supplement: FIGURE S2 — The stability of the VEGF responsive reporter-gene cell line was determined by testing VEGFA induced FL activity at regular intervals for 20 passages by incubating the VEGF responsive reporter-gene cells for 18 h with increasing concentrations of VEGFA prior to quantification of FL activity as described in the section “Materials and Methods”. [file Image_2.JPEG]

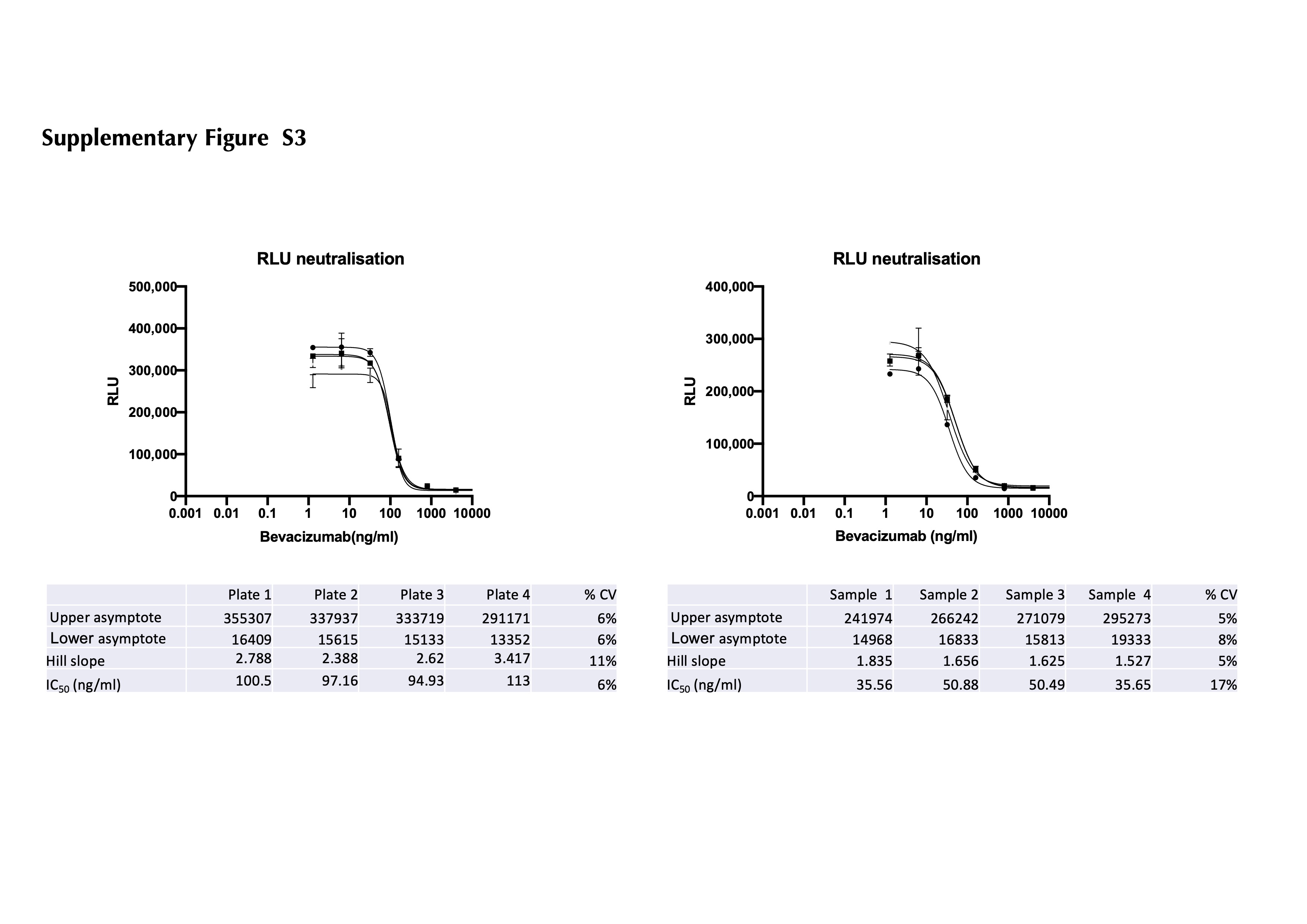

Supplement: FIGURE S3 — Inter and intra-assay precision was determined by incubating increasing concentrations of bevacizumab with 4 individual samples of VEGFA at a final concentration of 25 ng/ml for 30 min at room temperature on a single microtiter plate (A) or with a single sample of VEGFA at a final concentration of 25 ng/ml and incubated for 30 min at room temperature on 4 individual microtiter plates (B) prior to incubation for 18 h at 37°C with the VEGF responsive reporter-gene cell line and quantification of FL activity as described in the section “Materials and Methods.” The associated Table to Figure 2 shows the principal parameters of a 4PL plot determined using the Prism software together with intra-plate or inter-plate percentage coefficients of variation (% CV). [file Image_3.JPEG]

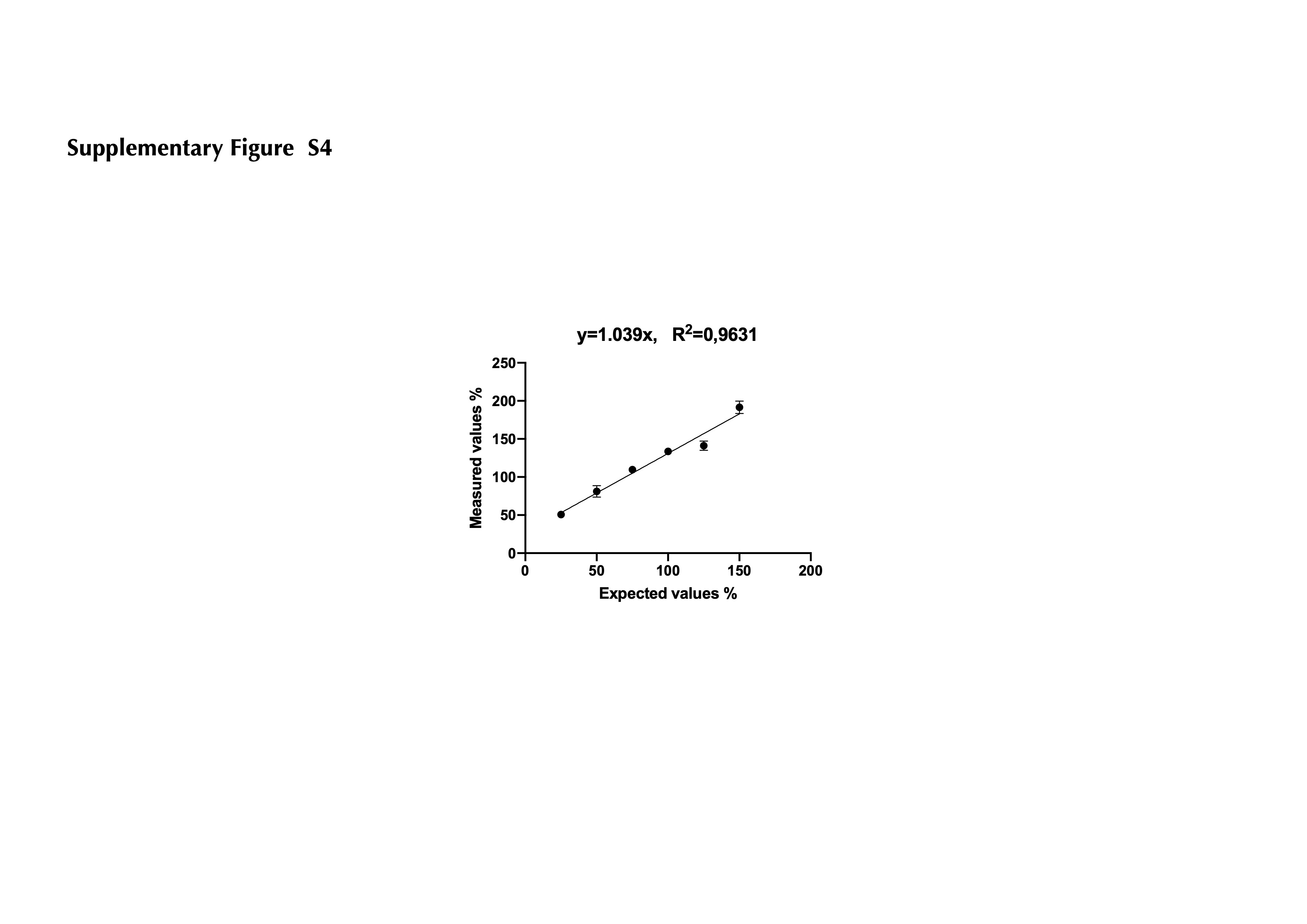

Supplement: FIGURE S4 — The accuracy and linearity of the reporter-gene assay was determined by incubating concentrations bevacizumab corresponding to 25, 50, 100, 125, and 150% of the expected value with VEGFA at a final concentration of 25 ng/ml for 30 min at room temperature prior to incubation for 18 h at 37°C with the VEGF responsive reporter-gene cell line and quantification of FL activity as described in the section “Materials and Methods”. [file Image_4.JPEG]

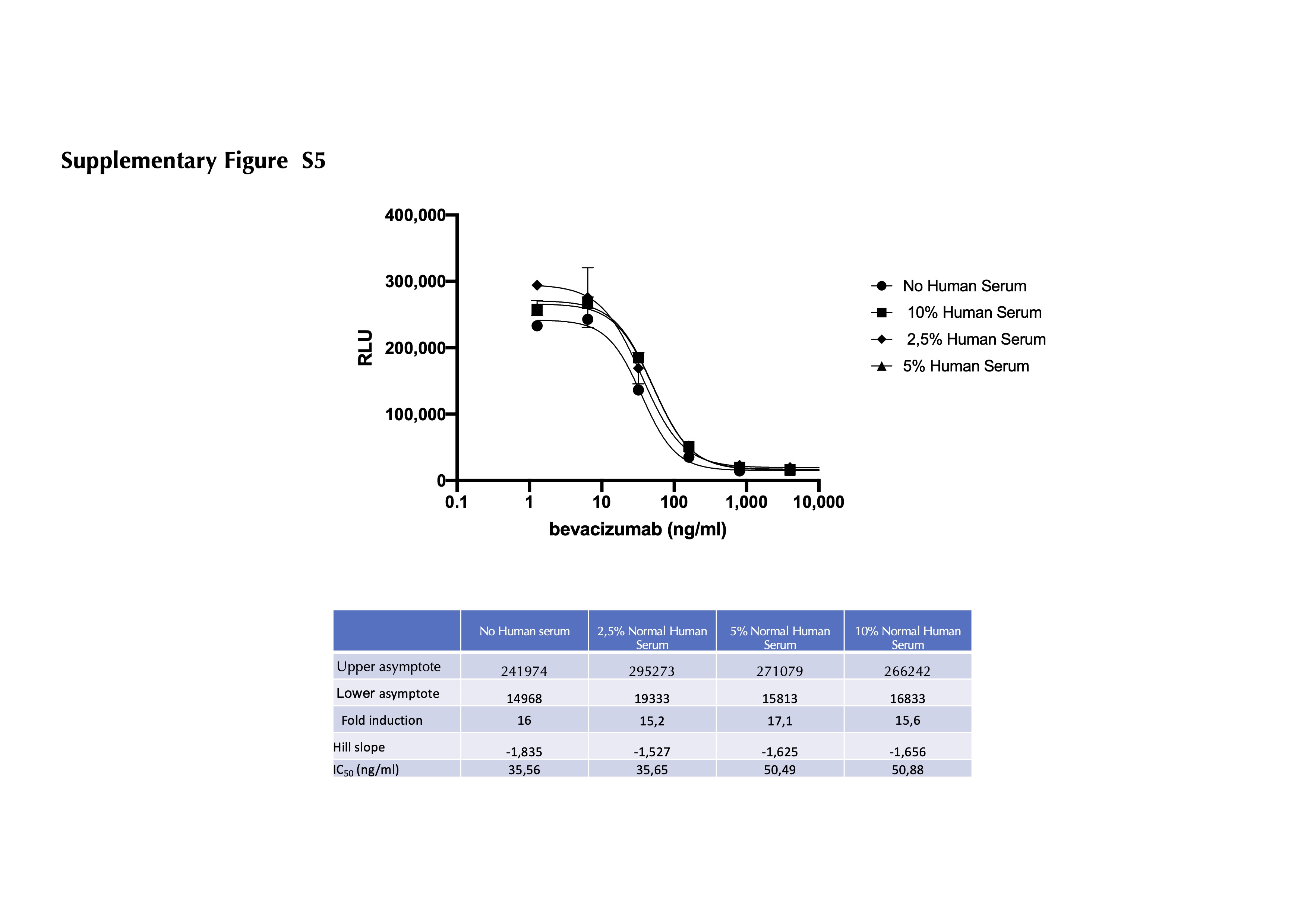

Supplement: FIGURE S5 — Increasing concentrations of bevacizumab were mixed with 25 ng/ml of VEGFA for 30 min at room temperature prior to incubation for 18 h with VEGF responsive reporter-gene cells either alone or in the presence of 2.5, 5, or 10% normal human serum and quantification of FL activity as described in the section “Materials and Methods” (B). The associated Table to Figure 1 shows the principal parameters of a 4PL plot determined using the Prism software. [file Image_5.JPEG]

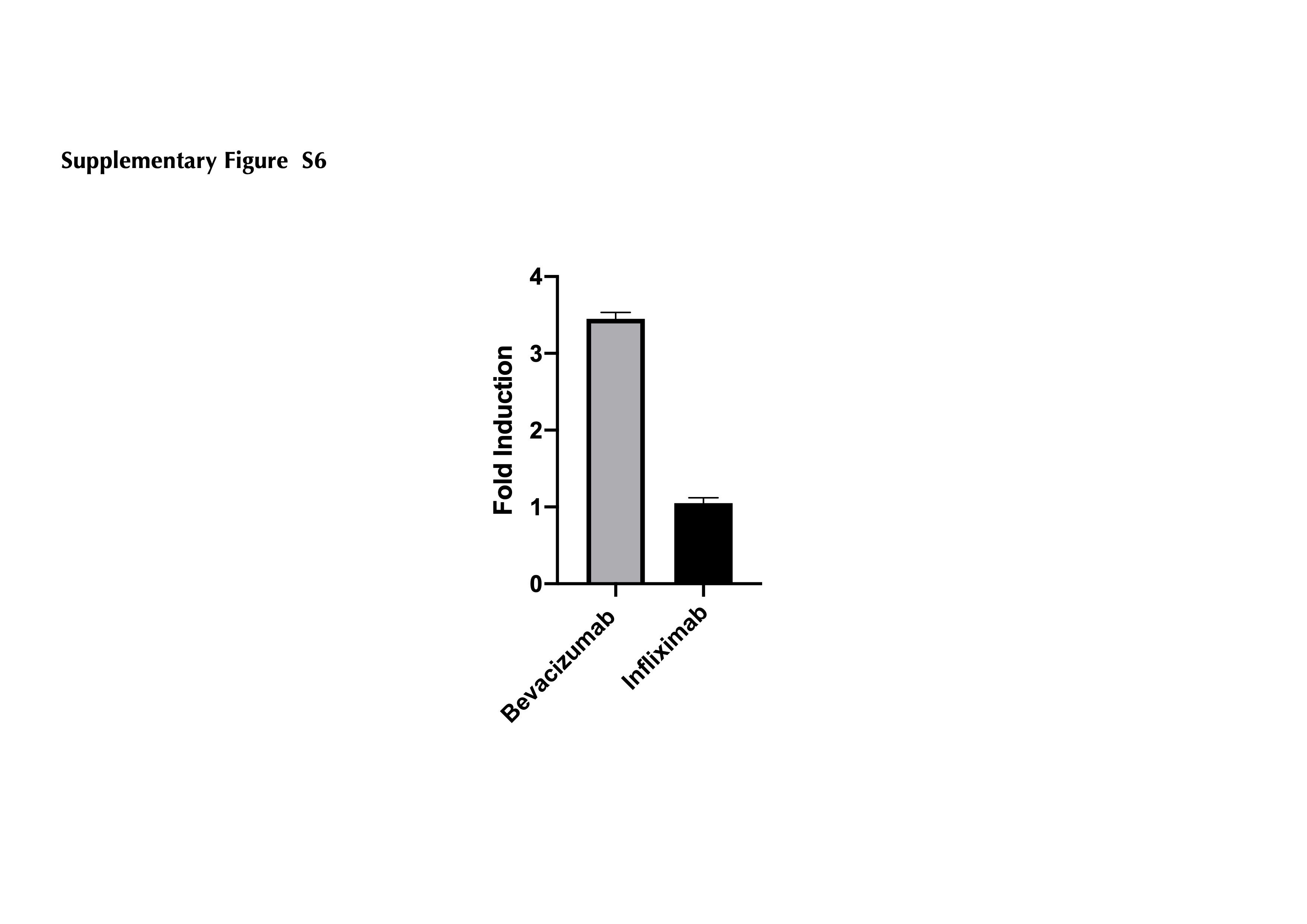

Supplement: FIGURE S6 — ADCC effector cells (E) at a concentration of (1.2 × 10^5^ cells/well), were incubated with human U87 glioblastoma target cells (T) at an E:T ratio of 3:1 and 100 μg/ml of bevacizumab or 100 μg/ml of inflixumab for 4 h prior to the quantification of ADCC activity as described in the section “Materials and Methods”. [file Image_6.JPEG]
