# Supplementary material for: Quantification of Bevacizumab Activity Following Treatment of Patients With Ovarian Cancer or Glioblastoma
Source: Front Immunol. 2020 Oct 15;11:515556. doi: 10.3389/fimmu.2020.515556 (PMC7593583; doi:10.3389/fimmu.2020.515556)
Supplement: TABLE S1 — Patients with ovarian cancer: Treatment schedule*. [file Table_1.docx]

**Supplementary Table S1**

**Patients with ovarian cancer: Treatment schedule***

| **Patient*** | **Sex** | **Age** | **Recent or Current use of Corticosteroids** | **Temodal** | **Radiotherapy** | **Other Previous Chemotherapy** |
| --- | --- | --- | --- | --- | --- | --- |
| IBK-101 | F | 67,3 | **No** | **No** | **No** | **Yes** |
| IBK-102 | F | 62,7 | **No** | **No** | **No** | **Yes** |
| IBK-103 | F | 57,7 | **No** | **No** | **No** | **Yes** |
| IBK-104 | F | 64,7 | **No** | **No** | **No** | **Yes** |
| IBK-105 | F | 70,7 | **No** | **No** | **No** | **Yes** |
| IBK-106 | F | 70,3 | **No** | **No** | **No** | **Yes** |
| IBK-107 | F | 37,9 | **No** | **No** | **No** | **Yes** |
| IBK-108 | F | 75,6 | **No** | **No** | **No** | **Yes** |
| IBK-109 | F | 61,2 | **No** | **No** | **No** | **Yes** |
| IBK-110 | F | 78,9 | **No** | **No** | **No** | **Yes** |
| IBK-111 | F | 52,7 | **No** | **No** | **No** | **Yes** |
| IBK-112 | F | 80,2 | **No** | **No** | **No** | **Yes** |
| IBK-113 | F | 73,2 | **No** | **No** | **No** | **Yes** |
| IBK-114 | F | 85,7 | **No** | **No** | **No** | **Yes** |
| IBK-115 | F | 73,4 | **No** | **No** | **No** | **Yes** |
| IBK-116 | F | 64,7 | **No** | **No** | **No** | **Yes** |
| IBK-117 | F | 72,9 | **No** | **No** | **No** | **Yes** |
| IBK-118 | F | 84,9 | **No** | **No** | **No** | **Yes** |
| IBK-119 | F | 40,4 | **No** | **No** | **No** | **Yes** |
| IBK-120 | F | 75,9 | **No** | **No** | **No** | **Yes** |
| IBK-121 | F | 62,5 | **No** | **No** | **No** | **Yes** |
| IBK-122 | F | 62,0 | **No** | **No** | **No** | **Yes** |
| IBK-123 | F | 64,5 | **No** | **No** | **No** | **Yes** |
| IBK-124 | F | 55,6 | **No** | **No** | **No** | **Yes** |
| IBK-125 | F | 37,4 | **No** | **No** | **No** | **Yes** |
| IBK-126 | F | 55,0 | **No** | **No** | **No** | **Yes** |
| IBK-127 | F | 69,0 | **No** | **No** | **No** | **Yes** |
| IBK-128 | F | 79,1 | **No** | **No** | **No** | **Yes** |
| IBK-129 | F | 44,7 | **No** | **No** | **No** | **Yes** |
| IBK-130 | F | 47,1 | **No** | **No** | **No** | **Yes** |
| IBK-131 | F | 46,6 | **No** | **No** | **No** | **Yes** |
| IBK-132 | F | 80,1 | **No** | **No** | **No** | **Yes** |
| IBK-133 | F | 69,9 | **No** | **No** | **No** | **Yes** |
| IBK-134 | F | 69,9 | **No** | **No** | **No** | **Yes** |
| IBK-135 | F | 72,7 | **No** | **No** | **No** | **Yes** |
| IBK-136 | F | 45,9 | **No** | **No** | **No** | **Yes** |
| IBK-137 | F | 81,2 | **No** | **No** | **No** | **Yes** |
| IBK-138 | F | 77,4 | **No** | **No** | **No** | **Yes** |
| IBK-139 | F | 75,8 | **No** | **No** | **No** | **Yes** |
| IBK-140 | F | 69,4 | **No** | **No** | **No** | **Yes** |
| IBK-141 | F | 47,8 | **No** | **No** | **No** | **Yes** |
| IBK-142 | F | 25,3 | **No** | **No** | **No** | **Yes** |
| IBK-143 | F | 54,8 | **No** | **No** | **No** | **Yes** |
| IBK-144 | F | 70,3 | **No** | **No** | **No** | **Yes** |
| IBK-145 | F | 82,0 | **No** | **No** | **No** | **Yes** |
| IBK-146 | F | 76,6 | **No** | **No** | **No** | **Yes** |
| IBK-147 | F | 59,1 | **No** | **No** | **No** | **Yes** |

*All patients with ovarian cancer were treated with 15 mg of bevacizumab per kg of body weight every 3 weeks as monotherapy.
